# Coronary Artery Disease in Cardiac Amyloidosis: Prevalence, Clinical Relevance, and Cardiac Magnetic Resonance Imaging Features

**DOI:** 10.3390/jcm14248802

**Published:** 2025-12-12

**Authors:** Carolina Donà, Renè Rettl, Christina Binder-Rodriguez, Daniel Dalos, Christina Kronberger, Michael Poledniczek, Robin Willixhofer, Nikita Ermolaev, Luciana Camuz-Ligios, Hermine Agis, Matthias Koschutnik, Dietrich Beitzke, Christian Loewe, Christian Nitsche, Christian Hengstenberg, Roza Badr Eslam, Johannes Kastner, Jutta Bergler-Klein, Andreas Anselm Kammerlander, Franz Duca

**Affiliations:** 1 Division of Cardiology, Department of Internal Medicine II, Medical University of Vienna, 1090 Vienna, Austria; carolina.dona@meduniwien.ac.at (C.D.); rene.rettl@meduniwien.ac.at (R.R.); christina.binder-rodriguez@meduniwien.ac.at (C.B.-R.); daniel.dalos@meduniwien.ac.at (D.D.); robin.willixhofer@meduniwien.ac.at (R.W.); nikita.ermolaev@meduniwien.ac.at (N.E.); luciana.camuz-ligios@meduniwien.ac.at (L.C.-L.); christian.nitsche@meduniwien.ac.at (C.N.); christian.hengstenberg@meduniwien.ac.at (C.H.); roza.badr-eslam@meduniwien.ac.at (R.B.E.); johannes.kastner@meduniwien.ac.at (J.K.); jutta.bergler-klein@meduniwien.ac.at (J.B.-K.); franz.duca@meduniwien.ac.at (F.D.); 2 Division of Hematology, Department of Internal Medicine I, Medical University of Vienna, 1090 Vienna, Austria; hermine.agis@meduniwien.ac.at; 3 Division of Cardiovascular and Interventional Radiology, Department of Bioimaging and Image-Guided Therapy, Medical University of Vienna, 1090 Vienna, Austria; dietrich.beitzke@meduniwien.ac.at (D.B.); christian.loewe@meduniwien.ac.at (C.L.)

**Keywords:** coronary artery disease, cardiac amyloidosis, magnetic resonance imaging, mortality

## Abstract

**Background/Objectives**: Cardiac amyloidosis (CA) as well as coronary artery disease (CAD) are both highly prevalent among the elderly. However, both the prevalence and risk factors associated with significant CAD among patients with CA, as well as potential outcome disparities, remain mainly unexplored. This study aimed to show the prevalence of CAD in patients with CA, as well as to assess outcomes and differences in late gadolinium enhancement (LGE) in comparison to patients with lone CA. **Methods**: We retrospectively assessed CA patients who underwent CAD assessment between 2013 and 2023. The primary endpoint was all-cause death. A subgroup underwent cardiac magnetic resonance imaging (CMR) with LGE assessment. **Results**: Of 255 consecutive patients with CA, 81 patients had significant CAD. Differences could be found with respect to age, sex, arterial hypertension, and hyperlipidemia. Significant differences in CMR features could only be found with respect to indexed left-ventricular end-diastolic volume, as well as left-ventricular mass. CAD-specific LGE was present only in 17.7% of patients with CAD, while most patients showed typical amyloid LGE, making a viability diagnosis difficult via CMR, especially in patients with end-stage CA. No differences in outcomes could be observed according to the prevalence of CAD. **Conclusions**: Concomitant obstructive CAD is highly prevalent among patients with CA. However, the presence of CAD does not influence patient outcomes. Furthermore, our data suggests that CAD viability testing by CMR might be complicated in patients with concomitant CA due to the high prevalence of amyloid-specific LGE.

## 1. Introduction

Cardiac amyloidosis (CA) is a protein-folding disorder where misfolded proteins (amyloid) deposit in the extracellular space of the myocardium. In approximately 98% of patients, CA is either caused by immunoglobulin light-chain amyloid (AL) or by transthyretin amyloid (ATTR) [1].

AL amyloidosis is a systemic disease, and affected patients often feature concomitant cardiac, renal, hepatic, and neurological involvement. The diagnosis of AL CA is performed via cardiac biopsy or extracardiac biopsy in combination with amyloidosis-typical changes in echocardiography or cardiac magnetic resonance imaging (CMR) studies [1]. ATTR amyloidosis is the most common type of CA and can be diagnosed non-invasively in the majority of patients using bone scintigraphy in combination with serum/urine immunofixation and quantification of free light chains [2,3].

In echocardiography, a significant myocardial hypertrophy can be observed as well as specific echocardiographic features such as the cherry-on-top sign, apical sparing or reduced diastolic dysfunction; to distinguish CA from other diseases causing hypertrophy, CMR is an important tool [4].

The deposition of amyloid leads to a progressive type of heart failure combined with myocardial hypertrophy, and if left untreated, is associated with a high mortality and morbidity. Due to the infiltration of the extracellular space, in CMR studies, an increase in extracellular volume (ECV) can be observed. Furthermore, patients with advanced CA often present with pathological patterns of late gadolinium enhancement (LGE) [5].

In addition to depositions in the myocardial extracellular space, amyloid can also deposit around coronary arteries, leading to myocardial ischemia or worsening of pre-existing coronary artery disease (CAD) [6]. However, thus far, data on the prevalence of CAD among CA patients is scarce. Furthermore, dedicated studies investigating the LGE pattern of CA patients with and without significant CAD are lacking [7].

The aims of this study are to assess the prevalence of the coexistence of CAD and CA, to examine differences in LGE patterns in CMR, and to compare outcomes between CA patients with and without CAD.

## 2. Materials and Methods

### 2.1. Study Design

This study was performed as part of the prospective amyloidosis registry at the Department of Cardiology at the Medical University of Vienna. The ethics committee of the Medical University of Vienna approved the study (EK No. 1079/2023); due to the retrospective nature of the study, written informed consent was waived. The study was performed according to good clinical practice set out by the Declaration of Helsinki.

### 2.2. Diagnostic Procedures

All patients underwent bone scintigraphy using ^99m^technetium-3,3-diphosphono-1,2-propanodicarboxylic acid (DPD) as the tracer. Furthermore, quantification of serum free light chains, as well as serum and urine protein electrophoresis and immunofixation, was performed in all study participants.

#### 2.2.1. Cardiac Transthyretin Amyloidosis

In patients who were included before publication of the non-biopsy algorithm for ATTR CA by Gillmore et al. in 2016 [2], or ambiguous non-invasive test results, the diagnosis was made via endomyocardial biopsy (EMB) and subsequent (immuno)-histological analysis of EMB specimens. In accordance with the current European Society of Cardiology (ESC) position paper regarding the diagnosis and treatment of CA, a non-invasive diagnosis of ATTR CA was made in patients with Perugini grade 2 or 3 and absence of paraprotein [1,2].

If EMB samples stained positive with Congo red and showed apple-green birefringence under polarized light, CA was confirmed. For subtyping, either immunohistochemistry (AmY-kit amyloid antibodies, Martinsried, Germany) or mass spectrometry was used [8]. Alternatively, CA could be diagnosed with extracardiac biopsy and imaging features for CA as outlined in the ESC position manuscript [1].

All patients with a diagnosis of ATTR amyloidosis were offered genetic testing to differentiate between wild-type (ATTRwt) and variant (ATTRv) transthyretin amyloidosis.

#### 2.2.2. Immunoglobulin Light-Chain Amyloidosis

AL CA was diagnosed if EMB samples stained positive for Congo red, showed apple-green birefringence under polarized light, and reacted with anti-AL (either kappa or lambda) antibodies. In cases of AL-positive extra-cardiac biopsy samples, cardiac involvement was defined via mean left-ventricular (LV) wall thickness (septum and posterior wall) > 12 mm in the absence of hypertension or other potential causes of LV hypertrophy in accordance with current recommendations from the International Symposium on Amyloid and Amyloidosis [9].

### 2.3. Outcome Measures

The primary endpoint for the present study was all-cause mortality. Endpoint data was compiled using (1) periodic queries on the national statistics authority’s (Statistik Austria) death registry, (2) routine out-patient visits, and (3) telephone interviews with patients or patients’ relatives.

### 2.4. Cardiac Magnetic Resonance Imaging

All CMR studies were performed on a 1.5 T system (MAGNETOM Avanto FIT; Siemens Healthineers, Erlangen, Germany) using standard protocols. Long-axis views and contiguous short-axis slice stacks (slice thickness: 6 mm, no gap, 25 phases/RR interval) were acquired with electrocardiography-gated steady-state free-precession cine images, while employing a breath-holding technique. LV and RV endocardial and epicardial contours were traced manually on the short-axis slice stacks, during end-systole and end-diastole, using dedicated software (Medis Medical Imaging, Medis Suite MR, Leiden, The Netherlands), to quantify LV and RV function and volumes. Furthermore, left-ventricular myocardial mass was assessed via the myocardial volume multiplied by the specific gravity of the myocardium (1.05 g/mL). Late gadolinium enhancement (LGE) was performed 10 min after application of a gadolinium-based contrast agent. The presence of LGE was assessed using the 17-segment model [10]; furthermore, the distribution, extent, as well as pattern of LGE were described according to respective guidelines [11].

T1 mapping sequences were generated before and 15 min after contrast agent application. Native T_1_-mapping was performed using electrocardiographically triggered MOLLI based on a 5(3)3 prototype (5 acquisition heartbeats are followed by 3 recovery heartbeats and a further 3 acquisition heartbeats) on a short-axis mid-cavity slice and a four-chamber view. For post contrast T1-mapping, a 4(1)3(1)2 prototype was used. ROIs were defined as left-ventricular myocardium without post-ischemic LGE; however, a non-ischemic pattern was included in the analysis [12]. Extracellular volume (ECV) was calculated using the following formula [13]:

$$\mathrm{ECV}\;=\;\left(1-\mathrm{hematocrit}\right)\;\times \;\frac{\left(\frac{1}{T1\;\mathrm{myo}\;\mathrm{post}}\right)-\;\left(\frac{1}{T1\;\mathrm{myo}\;\mathrm{pre}}\right)}{\left(\frac{1}{T1\;\mathrm{blood}\;\mathrm{post}}\right)-\left(\frac{1}{T1\;\mathrm{blood}\;\mathrm{pre}}\right)}$$

where “T1 myo pre”/”T1 blood pre” indicates myocardial/blood native T1 s and “T1 myo post”/“T1 blood post” indicates T1 times of myocardium/blood 15 min after contrast agent application. For the assessment of ECV, blood was drawn at the time of the CMR to assess the hematocrit.

All CMRs were evaluated by two blinded authors (A.K., C.D.), with each having more than 5 years of experience in CMR.

#### Assessment of Coronary Artery Disease

The presence of significant obstructive CAD was assessed in accordance with the 2019 ESC Guidelines for the diagnosis and management of chronic coronary syndromes, either non-invasively via cardiac computed tomography angiography (CCTA) or single-photon emission computed tomography (SPECT) perfusion imaging, or invasively via coronary angiography [14]. Furthermore, a history of myocardial infarction, coronary artery bypass graft (CABG), or percutaneous coronary intervention (PCI) was assessed at baseline. A diagnosis of CAD was confirmed if at least one of the following criteria was present: prior history of PCI and/or CABG or obstructive coronary artery stenosis of ≥70% by CCTA or invasive coronary angiography, or a ≥50% stenosis of the left main coronary artery. An exclusion of CAD was made when either SPECT imaging showed no significant ischemia (defined as a cut-off for ischemic area of ≤10% according to guidelines) or exclusion of any significant coronary artery stenosis was performed by either coronary angiography or stress-perfusion CMR [14].

### 2.5. Statistical Analysis

Continuous data are presented as mean ± standard deviation (SD) or median and interquartile ranges (IQR), while categorical variables are presented as numbers and percentages. Uni- and multivariable Cox regression analyses, along with Kaplan–Meier analysis using the log-rank test, were applied to assess the association of variables of interest with patient outcomes. Adjusted hazard ratios were obtained using a forward stepwise analysis including all variables that were significantly associated with the outcome in the univariable model. Significance levels for removal from and addition to the model were set at 0.10 and 0.05, respectively. The estimated glomerular filtration rate (eGFR) was calculated using the Cockcroft–Gault formula. The cohort was divided into two groups depending on the presence of significant CAD. Two-sided *p*-values of <0.05 were used to indicate statistical significance. SPSS 29.0 (IBM SPSS, Armonk, NY, USA) was used for all analyses. Figures were created using Biorender.com.

## 3. Results

**Figure 1:**
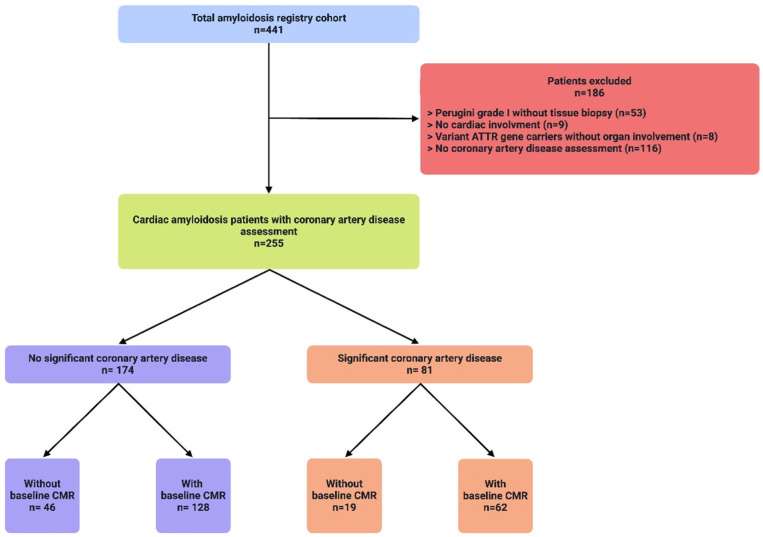
A total of 441 patients (blue) with cardiac amyloidosis (CA) were evaluated at the study center for the presence of coronary artery disease (CAD). A total of 186 patients (red) were excluded due to various reasons listed, the most common being a missing CAD assessment, as well as no histological confirmation in patients with Perugini grade I enhancement in DPD-scan. Of the remaining 255 patients (green), 174 patients (purple) had no signs of significant CAD according to current guidelines. A total of 180 patients underwent CMR with assessment of late gadolinium enhancement.

Of 441 patients in the Vienna amyloidosis registry between 2013 and 2023, 186 patients were excluded (see Figure 1—patient flow chart). In the remaining 255 patients with confirmed cardiac amyloidosis and screening for CAD, 81 patients (31.8%) had known significant CAD; out of those patients, 21 (25.9%) had a previous myocardial infarction, 61 (75.3%) had a previous PCI, and 14 (17.3%) had a previous CABG.

Significant differences between the groups (CAD vs. no CAD) could be found with respect to age (78.7 ± 7.3 vs. 75.5 ± 9.7 years, *p* = 0.011), sex (female patients 11.1% vs. 27.0%, *p* = 0.004), comorbidities such as arterial hypertension (77.8% vs. 54.6%, *p* < 0.001), and hyperlipidemia (59.3% vs. 36.8%, *p* < 0.001). N-terminal pro brain natriuretic peptide [(NT-proBNP) (median 3002 pg/mL; IQR 1523 to 6524 pg/mL vs. median 2991 pg/mL; IQR 1417 to 5523 pg/mL; *p* = 0.996)] did not show significant differences between the groups.

Though not statistically significantly different, AL CA was more prevalent than ATTR CA in patients without CAD (17.2% vs. 8.6%, *p* = 0.152). There were no statistically significant differences with respect to symptoms (NYHA ≥ II 84.0% vs. 88.5%, *p* = 0.678; CCS ≥ II 7.4% vs. 7.5%, *p* = 0.980), smoking status (16.0% vs. 9.8%, *p* = 0.128), or baseline LDL levels (74 ± 40 mg/dL vs. 87 ± 37 mg/dL, *p* = 0.863) (see Table 1).

### 3.1. Cardiac Magnetic Resonance Subgroup

Of the 255 patients, 190 (74.5%) underwent baseline CMR. Reasons for not performing CMR in the remaining 65 patients are depicted in Supplemental Table S1. CMR characteristics did not differ significantly between cohorts except for indexed left-ventricular end-diastolic volume (CAD vs. no CAD, 90.6 ± 20.4 mL/m^2^ vs. 81.7 ± 23.9 mL/m^2^, *p* = 0.014) and left-ventricular mass index (108 ± 28 g/m^2^ vs. 97 ± 26 g/m^2^, *p* = 0.034), which were higher in the CAD cohort. Functional parameters such as LVEF (49.0% vs. 51.5%, *p* = 0.183), RVEF (47.0% vs. 46.8%, *p* = 0.932), or ECV (46.0 ± 12.5% vs. 49.1 ± 13.2%, *p* = 0.130) did not differ significantly. Furthermore, native T1 times (1107 ± 87 ms vs. 1107 ± 73 ms, *p* = 0.885) were similar between cohorts (Table 2).

**Figure 2:**
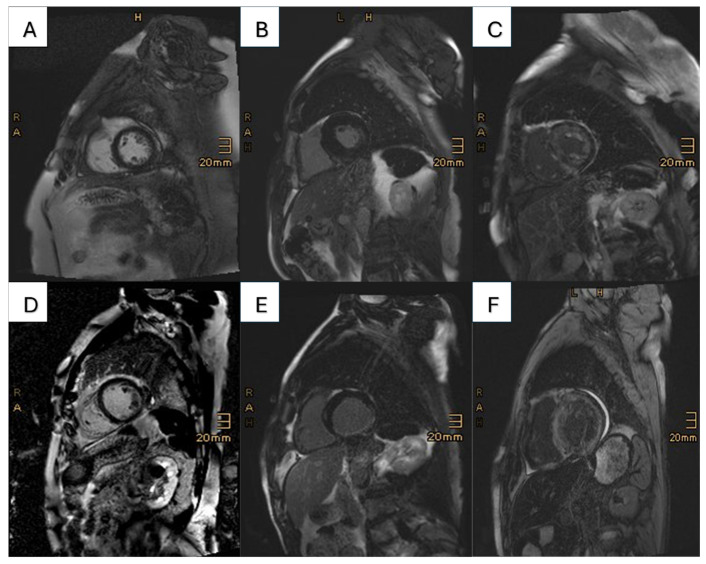
This figure presents the different LGE patterns prevalent in the cohorts: in the first row, patients with lone CA are shown, and the second row features patients with concomitant CA and CAD. (**A**) No known CAD, no LGE; (**B**) no known CAD, CAD-specific LGE (most likely MINOCA); (**C**) no known CAD, amyloid-specific LGE; (**D**) known CAD, no LGE; (**E**) known CAD, CAD-specific LGE in the inferior wall (RCA infarct); (**F**) known CAD, amyloid-specific LGE.

Two patients did not receive the contrast agent due to refusal and unwillingness to undergo peripheral venous cannulation. Among the 188 patients who received contrast agent, only 9 patients (4.7%) had no signs of LGE. A total of 158 patients (83.2%) had a LGE distribution typical for cardiac amyloidosis; however, in the CAD cohort, a typical amyloidosis LGE pattern was less often observed (CAD vs. no CAD 74.2% vs. 87.5%, *p* < 0.001). In 113 (60.1%) patients, a base-to-apex gradient could be observed. Of note, differences in the type of cardiac amyloidosis (ATTR: 70.4% vs. AL: 86.6%, *p* = 0.002) could be detected in patients with respect to amyloid-specific LGE patterns (Table 3). A total of 12 patients (6.3%) had a postischemic LGE pattern. Different LGE patterns in CA patients with and without significant CAD are depicted in Figure 2A–F. Only 1 of these patients had no previous diagnosis of CAD. In this patient, the LGE pattern was typical for a postischemic scar in the circumflex artery (CX). Of note, the coronary angiogram 14 days prior to CMR showed no signs of significant CAD. In the remaining patients with CAD-specific LGE pattern, four patients had LGE in the area of the CX, three patients had one in the left anterior descending artery (LAD) area, and three patients had postinfarction scar in the area of the right coronary artery (RCA). One patient showed a postischemic scar in the areas of both RCA and CX. Of the 11 patients with known CAD and postischemic pattern, seven had a previous myocardial infarction, three had a history of coronary artery bypass graft, and one patient showed diffusely calcified coronary arteries.

In four patients, an unspecific LGE (2.1%) was observed; in five (2.6%) patients, a LGE in the insertion points could be seen.

### 3.2. Outcome

**Figure 3:**
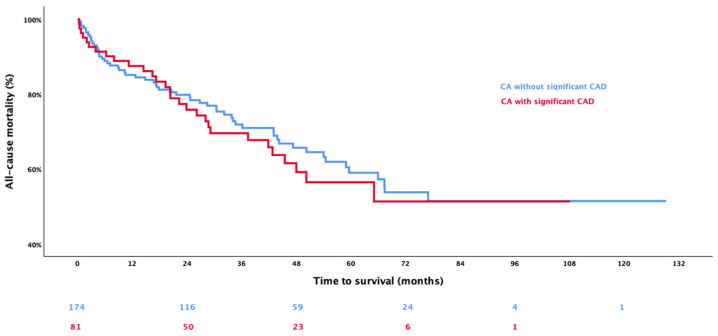
The Kaplan–Meier analysis shows the event-free survival in patients with cardiac amyloidosis (CA) stratified according to the presence of coronary artery disease (CAD), whereby red represents patients with concomitant CA and CAD, and blue represents those with lone CA.

After a mean follow-up time of 1147 ± 813 days, 89 deaths (34.9%) occurred (Supplemental Table S2). Kaplan–Meier curves and the respective log-rank test showed no significant difference between CA patients with CAD and without CAD (Figure 3, *p* = 0.636).

In the univariable Cox regression analysis, the presence of CAD was not significantly associated with worse patient outcomes (HR 1.056, 95% CI 0.667–1.671; *p* = 0.816), and neither were previous PCI (HR 1.044, 95% CI 0.631–1.725; *p* = 0.867), previous myocardial infarction (HR 0.587, 95% CI 0.238–1.449; *p* = 0.248), or CABG (HR 1.194, 95% CI 0.483–2.955; *p* = 0.701). Significant parameters associated with all-cause mortality were sex (female: HR 0.616, 95% CI 0.386–0.984, *p* = 0.042), diagnosis of ATTR CA (HR 0.319, 95% CI 0.195–0.521; *p* < 0.001), presence of carpal tunnel syndrome (HR 0.367, 95% CI 0.212–0.634; *p* < 0.001), hyperlipidemia (HR 0.527, 95% CI 0.328–0.848; *p* = 0.008), NT-proBNP (logarithmized; HR 3.220, 95% CI 2.000–5.185; *p* < 0.001), troponin T (HR 1.011, 95% CI 1.007–1.016; *p* < 0.001), eGFR (HR 0.984, 95% CI 0.975–0.993; *p* < 0.001), indexed LVEDV (HR 0.975, 95% CI 0.962–0.989; *p* < 0.001), and ECV (HR 1.022, 95% CI 1.000–1.044; *p* = 0.048).

In our multivariable Cox regression analysis, only a diagnosis of ATTR CA (HR 0.066, 95% CI 0.020–0.216; *p* < 0.001), troponin T (HR 1.010, 95% CI 1.005–1.015; *p* < 0.001), and eGFR (HR 0.969, 95% CI 0.950–0.988; *p* = 0.002) were significantly associated with outcome (Table 4).

The presence of any amyloid-specific or CAD-specific LGE pattern was not associated with outcome (HR 0.491, 95% CI 0.196–1.233, *p* = 0.130; HR 1.165, 95% CI 0.571–2.376, *p* = 0.675; HR 0.217, 95% CI 0.030–1.569, *p* = 0.130, respectively).

### 3.3. Timing of CAD Evaluation and Intervention

Forty-one (51.9%) of CA + CAD patients received their diagnosis of CAD over 1 year before that of CA, with a mean time prior to diagnosis of 3.2 ± 6.7 years, and 12 patients (15.2%) received the diagnosis within a year before the CA diagnosis. The remaining 26 patients (32.9%) were diagnosed with significant CAD after the confirmation of CA.

Of the 14 patients undergoing CABG, 13 patients (92.6%) were operated on over 1 year before CA diagnosis, and 1 patient (7.3%) underwent CABG after the diagnosis of ATTR amyloidosis.

Of the 61 patients with previous PCI, 23 (37.7%) underwent a coronary intervention after CA diagnosis; of those, 3 (13.0%) had a previously known CAD with worsening after diagnosis. A total of 8 (34.8%) patients underwent PCI within a year before CA diagnosis.

### 3.4. ECG Findings

Baseline ECGs of all patients were assessed. Mean limb voltage was 3.6 ± 1.5 mV, and mean precordial lead voltage was 6.9 ± 2.2 mV. There were no statistically significant differences between cohorts (CAD vs. no CAD 3.7 ± 14 vs. 3.5 ± 1.6 mV, *p* = 0.306; 7.3 ± 23 vs. 6.7 ± 2.1 mV, *p* = 0.060).

A pseudo-infarct pattern could be found in 21.1% of the patients without any statistically significant differences between CA patients with and without CAD (21.7% vs. 20.8%, *p* = 0.902).

## 4. Discussion

In our study of 255 CA patients, obstructive CAD was highly prevalent, with 31.7%. However, the presence of CAD had no influence on all-cause mortality or ventricular function. Furthermore, in the vast majority of cases, LGE patterns in patients with coexisting CAD and CA were amyloid-specific, rather than CAD-specific (Figure 2).

CA is now acknowledged as a clinically significant heart failure (HF) etiology that is also highly prevalent in patients with valvular heart disease (e.g., aortic stenosis and mitral regurgitation) [15,16]. However, only scarce data exist investigating the prevalence and clinical impact of CAD in this patient cohort.

Hassan et al. found a prevalence of 42% of CAD in ATTR patients, and in line with our results, their study also showed no outcome differences. However, in contrast to our results, their CAD cohort did not differ from the CAD-free cohort with respect to classic risk factors such as arterial hypertension and hyperlipidemia [17]. Furthermore, their study did not include patients with AL CA. Another study that analyzed coronary angiograms of HF patients with (*n* = 55) and without (*n* = 55) concomitant ATTR CA also found a similar CAD prevalence, with 30.9%. Moreover, clinical characteristics of ATTR + CAD patients were comparable to our cohort. However, patient outcomes were not assessed [18].

In line with previous studies, no differences in outcome between CA patients with and without CAD were observed (*p* = 0.636). These findings were reproducible even when the cohorts of ATTR and AL CA were analyzed separately. Risk factors for mortality in our study cohort were eGFR, troponin T and type of amyloidosis. Increased cardiac enzymes are known to be associated with a worse outcome both in chronic CAD patients [19,20], as well as CA [21,22]. Furthermore, it is known that patients with AL amyloidosis [23] tend to have a worse outcome in comparison to patients with TTR amyloidosis. Our study, therefore, confirms those findings in contemporary CA cohorts. Interestingly, the prevalence of CAD is lower in patients with AL amyloidosis, which can probably be explained by the generally younger population, lacking the classical risk factors for CAD. Further studies are needed to show any differences in CAD prevalence between AL and ATTR amyloidosis patients.

Thus far, studies evaluating CMR findings in patients with concomitant CAD and CA are lacking, although both entities have been studied extensively individually. In CA, a circular subendocardial to transmural LGE with increased native T1 times as well as increased ECV are typical findings [24], whereas in CAD, a subendocardial LGE restricted to areas of the supplying coronary arteries can be observed with partial thinning of the myocardium [25]. In our cohort, we could observe a high percentage of amyloid-specific changes, also in patients with concomitant CAD, as well as in patients with previous MI. CAD-specific changes could mainly be observed in patients with no or marginal LGE, or in areas that are typically less affected in CA (e.g., apical segments). Although further studies are needed, presumably with a comparative imaging technique showing myocardial viability, CMR might not be the ideal imaging modality, and alternative imaging techniques should likely be applied to provide a higher sensitivity. In our study, both cohorts showed significantly elevated ECV levels, a surrogate for myocardial amyloid burden; however, no differences between the groups could be observed [26]. This finding might be attributable to the exclusion of post-ischemic areas from the regions of interest for ECV quantification, as described in the Materials and Methods Section; however, when performing ECV analysis over the whole myocardium, including post-ischemic areas, no differences could be observed.

Among CMR characteristics, a higher left-ventricular end-diastolic volume in patients with CAD + CA could be observed. This is in accordance with former studies showing slightly increased end-diastolic volumes in patients with CAD [27,28].

No differences could be found with respect to ECG findings, making the pseudo-infarct pattern an unspecific sign of CA with no specificity for CAD.

In 2024, Chacko et al. showed severely reduced stress MRI perfusion in patients with CA, even worse in comparison to patients with known obstructive three-vessel disease [28]. Furthermore, similar baseline characteristics such as increased cardiac mass as well as increased indexed left-ventricular end-diastolic volume could be shown. However, CAD was evaluated only in a minority of patients. Therefore, undetected CAD could have influenced their findings in the CA cohort.

### Limitations

Several limitations of the present study need to be addressed. The single-center design may have led to a selection bias in our study population. Nonetheless, single-center studies have the advantage of constant quality of patient work-up, adherence to clinical routines, and follow-up. Furthermore, the generalizability of our CMR study results might be hampered by the fact that 65 patients of our CA cohort could not undergo CMR, especially considering that a high percentage of these patients had a device implanted, potentially creating a significant bias considering the severity of CA. However, baseline characteristics between patients undergoing CMR and those who could not did not show any significant differences other than the presence of a pacemaker (*p* value for all ≥ 0.05). A further limitation is that CAD was assessed using various techniques (invasive coronary angiography, computed tomography, myocardial scintigraphy) and not solely via the gold standard coronary angiography. Nonetheless, this diagnostic approach is reflective of real-world clinical practice and over half of all CAD diagnoses were made before enrolment into our CA registry. To rule out an immortal time bias, we performed a time-dependent Cox analysis, in which results remained unchanged; however, a potential bias due to the different time points of CAD diagnosis cannot be excluded. Furthermore, there are several baseline differences between the groups, especially the distribution of types of CA might present a relevant bias.

Last, but not least, one must admit that the study solely investigates the prevalence of epicardial atherosclerosis; however, the prevalence of microvascular amyloid-dependent angiopathy has not been explored by this study; further studies are needed to assess the prevalence of epicardial amyloid deposition and its influence on mortality.

## 5. Conclusions

Obstructive CAD in patients with CA is highly prevalent, irrespective of its subtype, and associated with classic risk factors such as arterial hypertension and hyperlipidemia. However, the presence of concomitant CAD in patients with CA does not influence patient outcomes. In our cohort of patients who underwent CMR, the presence of CAD was not associated with altered ventricular functions or LGE patterns. This suggests that CMR viability assessment may be of limited applicability in patients with CA; in patients with known CA and CAD with the need for viability assessment, alternative imaging techniques should be discussed.

## Figures and Tables

**Table 1:** Baseline characteristics: BMI—body mass index; CCS—Canadian coronary society; COPD—chronic obstructive pulmonary disease; CTS—carpal tunnel syndrome; eGFR—estimated glomerular filtration rate, Cockroft–Gault formula was used; ATTRv –variant ATTR amyloidosis; PCI—percutaneous coronary intervention; * only in patients with ATTR amyloidosis or combined ATTR and AL amyloidosis.

	Total (*n* = 255)	CAD (*n* = 81)	No CAD (*n* = 174)	*p*-Value
Age (years)	76.5 ± 9.1	78.7 ± 7.3	75.5 ± 9.7	**0.011**
Female patients (%)	56 (22.0)	9 (11.1)	47 (27.0)	**0.004**
Type of amyloidosis (%)				0.113
ATTR amyloidosis	216 (84.7)	74 (91.4)	142 (81.6)	
Of those ATTRv amyloidosis	17 (7.9)	5 (6.8)	12 (8.5)	
Of those, on treatment for ATTR amyloidosis	177 (82.7)	57 (77.0)	120 (84.5)	0.579
AL amyloidosis	37 (14.5)	7 (8.6)	30 (17.2)	
Of those, on treatment for AL amyloidosis	24 (64.9)	3 (42.9)	21 (70.0)	0.881
Combined ATTR and AL amyloidosis	2 (0.8)	0 (0.0)	2 (1.1)	
Of those, on treatment for amyloidosis	1 (50.0)	0 (0.0)	1 (50.0)	--
ATTR-score (%) *				0.227
I	93 (42.7)	30 (40.5)	63 (43.8)	
II	72 (33.0)	21 (28.4)	51 (35.4)	
III	53 (24.3)	23 (31.1)	30 (20.8)	
BMI (kg/m^2^)	26.0 ± 4.0	25.4 ± 3.4	26.2 ± 4.3	0.109
NYHA class ≥ II (%)	222 (87.1)	68 (84.0)	154 (88.5)	0.678
CCS ≥ II (%)	19 (7.5)	6 (7.4)	13 (7.5)	0.980
CTS (%)	96 (37.6)	30 (37.0)	66 (37.9)	0.996
Neurological symptoms (%)	142 (56.3)	40 (50.6)	102 (59.0)	0.216
Neurological symptoms in patients with ATTRv amyloidosis	12 (70.6)	5 (100.0)	7 (58.3)	0.086
Current smoker (%)	30 (11.8)	13 (16.0)	17 (9.8)	0.128
Diabetes mellitus (%)	54 (21.2)	23 (28.4)	31 (17.8)	0.054
Atrial fibrillation (%)	148 (58.0)	45 (55.6)	103 (59.2)	0.583
Arterial hypertension (%)	158 (62.0)	63 (77.8)	95 (54.6)	**<0.001**
Hyperlipidemia (%)	112 (43.9)	48 (59.3)	64 (36.8)	**<0.001**
COPD (%)	24 (9.4)	9 (11.1)	15 (8.6)	0.552
Coronary artery disease	81 (31.8)	81 (100.0)	0 (0.0)	**<0.001**
Previous myocardial infarction (%)	21 (8.2)	21 (25.9)	0 (0.0)	**<0.001**
Previous coronary artery bypass graft (%)	14 (5.5)	14 (17.3)	0 (0.0)	**<0.001**
Previous PCI (%)	61 (23.9)	61 (75.3)	0 (0.0)	**<0.001**
NT proBNP (pg/mL); median (IQR)	3002 (1460; 5799)	3002 (1523; 6524)	2991 (1417; 5523)	0.996
Troponin T (ng/L); median/IQR	51 (33; 81)	53 (37.5; 69.5)	49.5 (30; 90.5)	0.827
Creatine kinase (U/L)	110 ± 90	103 ± 53	113 ± 101	0.419
eGFR (mL/min)	58 ± 27	51 ± 21	61 ± 29	0.074
LDL (mg/dL)	83 ± 38	74 ± 40	87 ± 37	0.863
HbA1c (%)	5.9 ± 0.7	6.1 ± 0.8	5.9 ± 0.7	0.093

**Table 2:** CMR features: ECV—extracellular volume; EDD—end-diastolic diameter; EDV—end-diastolic volume; EF—ejection fraction; IVS—interventricular septum; LA—left atrium; LV—left ventricular; RA—right atrium; RV—right ventricular.

	Total (*n* = 190)	CAD (*n* = 62)	No CAD (*n* = 128)	*p*-Value
LVEDD (mm)	44.5 ± 7.0	46.6 ± 7.3	43.5 ± 6.6	**0.008**
RVEDD (mm)	39.8 ± 7.7	40.4 ± 8.2	39.5 ± 7.5	0.477
IVS (mm)	18 ± 4	19 ± 4	18 ± 4	0.702
Ascending aorta (mm)	35 ± 5	36 ± 4	35 ± 5	0.194
Pulmonary trunk (mm)	28 ± 5	27 ± 4	28 ± 5	0.159
LVEF (%)	50.7 ± 12.5	49.0 ± 13.5	51.5 ± 12.0	0.183
Indexed LVEDV (mL/m^2^)	84.5 ± 23.2	90.6 ± 20.4	81.7 ± 23.9	**0.014**
LV cardiac index (L/min/m^2^)	2.86 ± 0.74	2.91 ± 0.61	2.83 ± 0.80	0.543
LV mass index (g/m^2^)	100 ± 27	108 ± 28	97 ± 26	**0.034**
RVEF (%)	46.8 ± 11.4	47.0 ± 12.7	46.8 ± 10.9	0.932
Indexed RVEDV (mL/m^2^)	89.7 ± 24.1	89.3 ± 25.0	89.9 ± 23.8	0.872
RV cardiac index (L/min/m^2^)	2.79 ± 0.76	2.71 ± 0.61	2.82 ± 0.83	0.405
LA area (cm^2^)	32.0 ± 8.2	32.5 ± 8.4	31.7 ± 8.1	0.577
RA area (cm^2^)	30.2 ± 7.6	29.9 ± 7.6	30.3 ± 7.7	0.755
Pericardial effusion (%)	64 (35.2)	20 (34.5)	44 (35.5)	0.899
ECV (%)	48.1 ± 13.0	46.0 ± 12.5	49.1 ± 13.2	0.130
Native T1 times (ms)	1107 ± 77	1107 ± 87	1107 ± 73	0.885

**Table 3:** Late gadolinium enhancement, specific distribution; LGE—late gadolinium enhancement; base-to-apex gradient was only evaluated in patients with amyloid-specific LGE.

	Total (*n* = 190)	CAD (*n* = 62)	No CAD (*n* = 128)	*p*-Value
Type of LGE (%)				**<0.001**
No LGE	9 (4.7)	2 (3.2)	7 (5.5)	
Amyloid-specific LGE	158 (83.2)	46 (74.2)	112 (87.5)	
postischemic LGE	12 (6.3)	11 (17.7)	1 (0.8)	
Insertion points	5 (2.6)	2 (3.2)	3 (2.3)	
Unspecific LGE	4 (2.1)	1 (1.6)	3 (2.3)	
No contrast agent given	2 (1.1)	0 (0.0)	2 (1.6)	
Base-to-apex gradient (%)	113 (60.1)	35 (56.5)	78 (61.9)	0.382

**Table 4:** Uni- and multivariate Cox regression: BMI—body mass index; CCS—Canadian coronary society; COPD—chronic obstructive pulmonary disease; CTS—carpal tunnel syndrome; eGFR—estimated glomerular filtration rate, Cockroft–Gault formula was used; MI—myocardial infarction; PCI—percutaneous coronary intervention.

	Univariate Regression		Multivariate Regression	
Hazard Ratio (95%CI)		*p*-Value	Hazard Ratio (95% CI)	*p*-Value
Baseline Characteristics				
Age	1.015 (0.989–1.042)	0.258		
Gender (female patients)	0.616 (0.386–0.984)	**0.042**		
Type of amyloidosis (AL = 0, aTTR = 1)	0.319 (0.195–0.521)	**<0.001**	0.066 (0.020–0.216)	**<0.001**
BMI	0.959 (0.906–1.015)	0.149		
NYHA class ≥ II	3.726 (1.176–11.805)	**0.025**		
CCS ≥ II	1.669 (0.766–3.635)	0.197		
CTS	0.367 (0.212–0.634)	**<0.001**		
Current smoker	0.721 (0.332–1.566)	0.408		
Diabetes mellitus	1.078 (0.623–1.864)	0.789		
Atrial fibrillation	1.127 (0.728–1.745)	0.591		
Arterial hypertension	0.761 (0.494–1.172)	0.215		
Hyperlipidemia	0.527 (0.328–0.848)	**0.008**		
COPD	2.150 (1.133–4.080)	**0.019**		
Coronary artery disease	1.056 (0.667–1.671)	0.816		
Previous MI	0.587 (0.238–1.449)	0.248		
Previous PCI	1.044 (0.631–1.725)	0.867		
Previous CABG	1.194 (0.483–2.955)	0.701		
**Laboratory parameters**				
proBNP (logarithmized)	3.220 (2.000–5.185)	**<0.001**		
Troponin T (logarithmized)	1.011 (1.007–1.016)	**<0.001**	1.010 (1.005–1.015)	**<0.001**
eGFR (ml/min)	0.984 (0.975–0.993)	**<0.001**	0.969 (0.950–0.988)	**0.002**
**CMR parameters**				
LVF (%)	1.011 (0.989–1.032)	0.334		
Indexed LVEDV (mL/m^2^)	0.975 (0.962–0.989)	**<0.001**		
RVEF (%)	0.990 (0.969–1.012)	0.369		
Indexed RVEDV (mL/m^2^)	0.992 (0.980–1.002)	0.214		
ECV (%)	1.022 (1.000–1.044)	**0.048**		
Presence of any LGE	0.491 (0.196–1.233)	0.130		
Presence of amyloid-specific LGE	1.165 (0.571–2.376)	0.675		
Presence of CAD-specific LGE	0.217 (0.030–1.569)	0.130		

## Data Availability

Data is available upon request.

## Supplementary Materials

The following supporting information can be downloaded at: https://www.mdpi.com/article/10.3390/jcm14248802/s1, Table S1: reasons for patients not undergoing CMR; Table S2: outcome assessment.

## Abbreviations

The following abbreviations are used in this manuscript:

AL	immunoglobulin light-chain amyloid
ATTR	transthyretin amyloid
CA	cardiac amyloidosis
CABG	coronary artery bypass graft
CAD	coronary artery disease
DPD	^99m^technetium-3,3-diphosphono-1,2-propanodicarboxylic acid
ECV	extracellular volume
LGE	late gadolinium enhancement
PCI	percutaneous coronary intervention
