# Supplementary material for: Coronary Artery Disease in Cardiac Amyloidosis: Prevalence, Clinical Relevance, and Cardiac Magnetic Resonance Imaging Features
Source: J Clin Med. 2025 Dec 12;14(24):8802. doi: 10.3390/jcm14248802 (PMC12734079; doi:10.3390/jcm14248802)
Supplement: Supplementary file 1 [file jcm-14-08802-s001.zip › jcm-3976309-supplementary.pdf]

Supplemental Table S1.

Reasons for patients not undergoing CMR.

44 patients	pacemaker in situ
5 patients	chronic kidney failure prohibiting contrast agent injection
5 patients	refusal of the patient
2 patients	claustrophobia
1 patient	death prior to CMR

Supplemental Table S2. Outcome assessment

	Total (n=255)	CAD (n=81)	No CAD (n=174)	p-Value
Follow-up time	1147 ± 813	1090 ± 737	1173 ± 847	0.144
All-cause mortality (%)	89 (34.9)	29 (35.8)	60 (34.5)	0.837
Cardiovascular cause (%)	39 (43.8)	11 (37.9)	28 (46.7)	
Unknown cause (%)	36 (40.4)	10 (34.5)	26 (43.3)	
